# Dr. Nathaniel Ware Hawes

**Published:** 1900-07

**Authors:** 


### DR. NATHANIEL WARE HAWES.

Dr. Nathaniel Ware Hawes, of Wrentham, Mass., one of the earliest active members of this Society, died at his residence in that town on Sunday morning, April 1, 1900.

Dr. Hawes was an honored member of the dental profession, and had practised dentistry in Boston since 1865. He was born in Wrentham August 12, 1838, in the same house in which he died. He was educated at Day's Academy in Wrentham, and studied his profession with Dr. George E. Hawes, of New York City. After this he graduated from the Harvard Dental School, and for a time practised dentistry in Wrentham and Foxboro. He was a demonstrator in operative dentistry, and was afterwards appointed assistant professor in the same chair in the school from which he graduated. Dr. Hawes contributed valuable papers to the literature of his profession, and was prominent in Masonic circles, as well as in public affairs in his native town. He was a man of great generosity and unlimited geniality of disposition. His unbounded hospitality is best known to us by the delightful outings that we had with him in his beautiful home in Wrentham. We shall miss his familiar face and the hearty grasp of his hand.

Whereas, It has pleased our Heavenly Father to take from us our honored member, Dr. Nathaniel Ware Hawes.

*Resolved,* That we, the Boston Society for Dental Improvement, desire to place upon our record our high appreciation of his professional standing; that we mourn his death as a personal as well as a professional loss, and desire to testify to our admiration for his efforts given at all times to advance the profession of his choice.

*Resolved,* That a copy of these resolutions be sent to his family, that they be spread upon the minutes of our Society, and that a copy be sent to the professional journals.

R. R. Andrews,
F. M. Robinson,
*Committee.*
H. S. Draper,
*Secretary.*

Boston, May 15, 1900.

